# The Impact of Antiretroviral Therapy on Electrocardiographic Parameters in Human Immundeficiency Virus‐Positive Patients

**DOI:** 10.1111/anec.70058

**Published:** 2025-03-25

**Authors:** Ahmet Anıl Başkurt, Yusuf Demir, Oktay Şenöz

**Affiliations:** ^1^ Department of Cardiology Bakırçay University Izmir Turkey

**Keywords:** antiretroviral therapy, electrocardiogram, human immundeficiency virus

## Abstract

**Background:**

Antiretroviral therapy (ART) has revolutionized the management of human immunodeficiency virus (HIV) infection by transforming it into a chronic but manageable condition. Despite its effectiveness in viral suppression and immune restoration, concerns remain regarding ART's potential impact on cardiovascular health, particularly on electrocardiographic (ECG) parameters.

**Objective:**

This study investigated the effects of ART on ECG parameters in HIV‐infected patients by analyzing pre‐ and post‐therapy data.

**Methods:**

A total of 83 HIV‐positive patients were enrolled and evaluated for ECG parameters before and 3 months after ART initiation. Key parameters, including QRS duration, QT duration corrected by the Bazett formula (QTc interval), QRS‐T angle, morphology in inferior leads, voltage in lead 1, and P‐wave duration (MVP) score, were manually assessed. Statistical analyses compared pre‐ and post‐ART values.

**Results:**

No statistically significant changes were observed in ECG parameters post‐ART. For example, QRS duration remained stable (pre‐ART: 89.08 ± 12.01 ms; post‐ART: 88.94 ± 10.00 ms, *p* = 0.849), as did QTc interval (pre‐ART: 403.51 ± 22.22 ms; post‐ART: 404.84 ± 14.91 ms, *p* = 0.563) and MVP ECG score (pre‐ART: 3.02 ± 0.95; post‐ART: 2.98 ± 0.87, *p* = 0.882). The QRS‐T angle also showed no significant difference (*p* = 0.675).

**Conclusion:**

ART does not appear to significantly affect ECG parameters in HIV‐infected patients, supporting its favorable cardiac safety profile. These findings highlight the importance of regular ECG monitoring to ensure cardiovascular safety in patients undergoing ART.

## Graphical abstract

In summary, the study findings suggest that ART does not significantly alter ECG parameters, including interatrial conduction, MVP ECG score, or QRS‐T angle. These results support the notion that ART has a favorable cardiac safety profile, with no measurable impact on cardiac electrical activity as assessed by ECG.

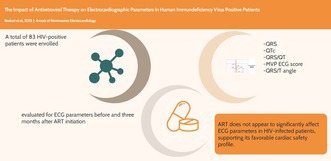

## 1 Introduction

Human immunodeficiency virus (HIV) remains a major global health issue, affecting millions of individuals worldwide. With the advancement of antiretroviral therapy (ART), HIV infection has transitioned from a fatal disease to a manageable chronic condition, significantly extending life expectancy in infected individuals (Barbara et al. 2016). However, despite the clinical benefits of ART in viral suppression and immune function restoration, there is increasing concern regarding its long‐term impact on cardiovascular health, specifically on cardiac electrical activity and electrocardiographic (ECG) parameters (Paternò Raddusa et al. 2024; Freiberg et al. 2017).

ART has been associated with a range of metabolic and cardiovascular complications, such as dyslipidemia, insulin resistance, and increased risk for myocardial infarction (MI) (Feinstein et al. 2019; Jain et al. 2001). These cardiovascular effects have led researchers to question whether ART might also influence ECG parameters, which serve as non‐invasive markers of cardiac electrical function and are crucial for identifying arrhythmias and other cardiac abnormalities. However, findings in the literature have been inconsistent, with some studies suggesting significant alterations in ECG metrics post‐ART, while others report negligible effects (Wongcharoen et al. 2014).

This study aimed to examine the impact of ART on ECG parameters in HIV‐infected patients by comparing pre‐ and post‐therapy data. We hypothesize that ART does not produce significant changes in ECG values, implying minimal direct effects of ART on cardiac electrical activity. Through this analysis, we seek to provide a clearer understanding of ART's impact on the heart's electrical function and contribute to the safe management of cardiovascular health in HIV patients undergoing ART.

## 2 Methods

### 2.1 Study Design and Ethical Approval

This study was conducted following the approval of Bakırçay University Non‐Invasive Ethics Committee (Approval date: November 20, 2024, Approval No:1777). The research included 83 patients diagnosed with HIV who presented to the cardiology outpatient clinic for evaluation prior to the initiation of ART. All participants were informed about the study, and written informed consent was obtained from each individual.

### 2.2 Study Population

The study population comprised 83 HIV‐positive patients who were evaluated both at baseline (prior to ART initiation) and during the follow‐up period. Patients were recalled for a follow‐up visit at the third month after starting ART. During each visit, comprehensive assessments were conducted, including anamnesis, a 12‐lead electrocardiogram (ECG), and transthoracic echocardiographic evaluation. Patients with any pre‐existing cardiovascular diseases, arrhythmias, or other conditions that could interfere with ECG parameters were excluded from the study. Although the duration of HIV infection is unknown, patients who were diagnosed within the last 6 months and accepted ART treatment were included in the study. In the treatment, personalized treatment has been started for the patients, and 81.9% of the patients are receiving emtricitabine/tenofovir disoproxil + dolutegravir treatment. Other patients receive lamivudine + dolutegravir, bictegravir/emtricitabine/tenofovir alafenamide treatments.

### 2.3 ECG Evaluation

Standard 12‐lead ECG recordings were obtained for all participants at a paper speed of 25 mm/s and a calibration of 10 mm/mV. The ECG parameters measured included QRS duration, corrected QT interval (QTc), QRS‐T angle, and morphology in inferior leads, voltage in lead 1, and P‐wave duration (MVP) score. All measurements were performed manually by two independent cardiologists blinded to the patients' treatment status.

The QRS duration is measured by identifying the beginning of the Q wave and the end of the S wave on the ECG trace. This interval reflects the time required for ventricular depolarization.

The corrected QT interval (QTc) is determined by measuring the QT interval, which starts at the beginning of the Q wave and ends at the end of the T wave. To account for variations in heart rate, the QT interval is corrected using formulas like Bazett's formula, where QTc equals the QT interval divided by the square root of the RR interval (measured in seconds). The RR interval represents the time between two consecutive R waves (Bazett 1997).

The QRS‐T angle is calculated by comparing the electrical axis of ventricular depolarization (represented by the QRS complex) with that of ventricular repolarization (represented by the T wave). This angle is often determined using the spatial relationship provided by the ECG machine. It represents the difference in the directional vectors of these electrical activities (Andršová et al. 2022).

The MVP score was comprised of three P‐wave variables: morphology in inferior leads, voltage in lead 1, and MVP Table 1 illustrates the MVP ECG risk score variables (Alexander et al. 2019).

**TABLE 1:** Demographic and baseline laboratory data.

Parameter	Value
Sample size	83
Mean age (years)	42.13 ± 11.96
Gender (male)	12 (14.5%)
Hypertension (%)	8.4%
Diabetes mellitus (%)	13.3%
Hyperlipidemia (%)	6.0%
Smoking status (smokers/non‐smokers)	67.9%/32.1%
Emtricitabine/tenofovir disoproxil + dolutegravir treatment (%)	81.9%
Fasting glucose (mg/dL)	93.41 ± 15.93
Creatinine (mg/dL)	0.89 ± 0.28
Uric acid (mg/dL)	5.44 ± 1.28
White blood Cell (×10^3^/μL)	6.59 ± 2.78
Hemoglobin (g/dL)	13.75 ± 2.13
C‐Reactive protein (mg/L)	8.53 ± 32.46

### 2.4 Statistical Analysis

Data were analyzed using SPSS version 22. Continuous variables were presented as mean ± standard deviation (SD), and categorical variables were expressed as frequencies and percentages. Comparisons between baseline and post‐ART values were performed using paired *t*‐tests for normally distributed variables and Wilcoxon signed‐rank tests for non‐normally distributed variables. A *p*‐value of < 0.05 was considered statistically significant. Interobserver variability for ECG measurements was assessed using intraclass correlation coefficients (ICCs), with values > 0.80 indicating excellent agreement.

## 3 Results

This study included a total of 83 HIV‐positive patients. 81.9% of the patients are receiving emtricitabine/tenofovir disoproxil + dolutegravir treatment. Other patients receive lamivudine + dolutegravir, bictegravir/emtricitabine/tenofovir alafenamide treatments. The mean age of the participants was 42.13 ± 11.96 years. The cohort consisted of 12 males (14.5%) and 71 females (85.5%). Among the participants, the prevalence of hypertension was 8.4%, diabetes mellitus was 13.3%, and hyperlipidemia was 6%. Additionally, smoking was reported by a significant proportion of the cohort (67.9%), while 32.1% were non‐smokers (Table 1).

The comparison of ECG parameters before and after ART initiation showed no statistically significant changes. The mean QRS duration was 89.08 ± 12.01 ms before ART and 88.94 ± 10.00 ms after ART (*p* = 0.849). Similarly, the QTc interval remained stable, with a mean of 403.51 ± 22.22 ms pre‐ART and 404.84 ± 14.91 ms post‐ART (*p* = 0.563). The QRS/QT ratio was unchanged, averaging 0.22 ± 0.03 in both pre‐ and post‐ART periods (*p* = 0.565). MVP ECG scores also showed no significant differences, with pre‐ART values of 3.02 ± 0.95 and post‐ART values of 2.98 ± 0.87 (*p* = 0.882). Finally, the QRS‐T angle showed a slight increase from 32.1° ± 10.2° pre‐ART to 33.5° ± 11.4° post‐ART, but this difference was not statistically significant (*p* = 0.675) (Table 2).

**TABLE 2:** ECG parameters pre‐ and Post‐ART.

Parameter	Pre‐ART (mean ± SD)	Post‐ART (mean ± SD)	*p*
QRS duration (ms)	89.08 ± 12.01	88.94 ± 10.00	0.849
QTc interval (ms)	403.51 ± 22.22	404.84 ± 14.91	0.563
QRS/QT ratio	0.22 ± 0.03	0.22 ± 0.03	0.565
MVP ECG score	3.02 ± 0.95	2.98 ± 0.87	0.882
QRS‐T angle (°)	32.1 ± 10.2	33.5 ± 11.4	0.675

## 4 Discussion

This study aimed to evaluate the impact of ART on ECG parameters in HIV‐infected patients. Our findings revealed no statistically significant differences in ECG parameters before and after ART initiation, suggesting that ART may not adversely affect cardiac electrical activity in this patient population.

Our results are consistent with existing literature on the cardiovascular effects of ART. For instance, Barbaro (2001) reported that while ECG abnormalities were observed in HIV‐infected patients, these were not significantly exacerbated by ART.

Our study differs from other research that often compares HIV‐positive patients with healthy individuals. For example, studies such as (Wongcharoen et al. 2014; Befkadu et al. 2024; Knudsen et al. 2019) showed that HIV‐positive individuals have a higher prevalence of QT prolongation and other ECG abnormalities compared to healthy controls. These studies suggest that systemic inflammation, autonomic dysfunction, or direct viral effects may contribute to these differences. In contrast, our within‐subject comparison of pre‐ and post‐ART ECG parameters focuses on ART's specific impact and reveals no significant changes, indicating that modern ART regimens are unlikely to exacerbate these abnormalities.

In the study conducted by Wu et al. (2019) QTc was statistically significantly higher in HIV (+) men than in HIV (−). In our study, gender differences were not taken into account. In this study, no relationship was found between ART and QTc, like our study, but high inflammatory markers were associated. Therefore, it is important to control viral replication.

Additionally, our findings align with previous research on the QRS‐T angle, a predictor of cardiovascular risk. The SMART study demonstrated that a widened QRS‐T angle was associated with cardiovascular events in HIV‐positive individuals (Dawood et al. 2013). In our study, no significant differences in the QRS‐T angle were observed pre‐ and post‐ART, reinforcing the notion that ART may not have a detrimental impact on ventricular repolarization. This supports the cardiovascular safety of contemporary ART regimens. In a cross‐sectional study in Nigeria, patients receiving ART had more ECG changes than patients not receiving ART and HIV‐negative patients. However, this study was a cross‐sectional study, and patients were not compared before and after ART. As baseline ECG data are unknown, the difference cannot be attributed to ART (Njoku et al. 2016).

In recent years, interatrial block and MVP risk scoring have become important in the ECG risk assessment of cardiovascular diseases, especially atrial fibrillation. (Alexander et al. 2019, 2017; de Luna et al. 2012; O'Neal et al. 2016; Tse et al. 2018) Studies have reported that interatrial block is more common in infected patients. (Russo et al. 2022; Cetin Guvenc et al. 2023) In the study conducted by Fanjul et al. (2019) interatrial block was found to be more common in HIV (+) patients in the presence of comorbidities and in the elderly. Although the CD4 counts were lower, there was no difference in the CD4/CD8 ratio. The treatment received by the patients was not mentioned in this study. Considering that inflammation in HIV (+) patients is systemic and one of the most important parameters that trigger atrial fibrillation is inflammation in the left atrium, it is thought that the frequency of atrial fibrillation will be higher in these patients (Elnahar et al. 2012). MVP risk scoring has been shown to predict the occurrence of atrial fibrillation in the long term in patients with high comorbidities (del Pilar Falcón et al. 2023; Pay et al. 2023). It is thought that ART, which suppresses this inflammation, may reduce the frequency of atrial fibrillation. In our study, no difference was observed in the MVP risk score before and after ART treatment, and since we had a relatively short follow‐up period for the occurrence of atrial fibrillation, it is necessary to wait for long‐term results.

The absence of significant ECG changes in our study may reflect several factors. First, modern ART regimens are optimized to minimize cardiovascular side effects, making adverse ECG alterations less likely. Second, the duration of ART exposure in our study may have been insufficient to detect measurable ECG changes, particularly those that might emerge over longer follow‐up periods. Lastly, individual patient characteristics, including genetic predispositions and the use of concurrent medications, could mitigate or mask the cardiac effects of ART.

Our findings are reassuring for clinicians managing HIV‐infected patients, as they suggest that the benefits of ART in controlling HIV replication and enhancing immune function are not offset by detrimental effects on cardiac electrophysiology. Nonetheless, continuous ECG monitoring remains crucial, particularly for patients with pre‐existing cardiovascular conditions or those receiving ART regimens with potential effects on cardiac conduction.

### 4.1 Limitations

This study has several limitations. The sample size was relatively small, which may limit the generalizability of our findings. Additionally, the follow‐up period may not have been long enough to detect late‐onset cardiac effects of ART. Future studies with larger cohorts and extended follow‐up durations are warranted to confirm our findings and explore the long‐term cardiac safety of various ART regimens.

## 5 Conclusion

In conclusion, our study indicates that ART does not significantly alter ECG parameters in HIV‐infected patients, suggesting a favorable cardiac safety profile. These findings contribute to the growing body of evidence supporting the cardiovascular safety of ART and underscore the importance of regular pre‐treatment cardiac monitoring in this patient population.

## Author Contributions

Conception and design of the research: A.A.B., O.Ş.; acquisition of data: Y.D., A.A.B., O.Ş.; analysis and interpretation of the data: A.A.B.; statistical analysis: A.A.B., O.Ş.; writing of the manuscript: A.A.B.; reviewed article: D.Y., O.Ş., A.A.B.

## Conflicts of Interest

The authors declare no conflicts of interest.

## Data Availability

The data that support the findings of this study are available from the corresponding author upon reasonable request.
